# Long-term results of cementless hip arthroplasty with ceramic-on-ceramic articulation

**DOI:** 10.1007/s00264-012-1639-x

**Published:** 2012-08-19

**Authors:** Marek Synder, Marek Drobniewski, Marcin Sibiński

**Affiliations:** Clinic of Orthopedic and Pediatric Orthopedics, Medical University of Lodz, Drewnowska 75, 91-002 Lodz, Poland

## Abstract

**Purpose:**

The goal of the study was to evaluate long-term results of hip arthroplasty in patients with ceramic-on-ceramic articulation.

**Methods:**

The follow-up involved 220 primary total hip arthroplasty procedures (188 patients, 101 women and 87 men) after implantation of the Mittelmeier cementless hip endoprosthesis. The mean age of patients at surgery was 44.5 years and the mean follow-up was 19.6 years, with a minimum of 12.3 years. Dysplastic, idiopathic and post-traumatic coxarthrosis were the most frequent forms of degenerative hip changes. The Merle d’Aubigné and Postel classification, as modified by Charnley, was used for clinical evaluation.

**Results:**

Very good results were obtained in 39.5 % of the patients, good results in 43.6 %, satisfactory results in 9.1 % and poor results in 7.8 %. Twelve-year survival for the whole prosthesis was 86.36 %, for the acetabulum 89.99 % and for the stem 91.36 %.

**Conclusions:**

Long-term results of hip arthroplasty using the Mittelmeier prosthesis are fairly encouraging with their low incidence of loosened prosthesis components after surgery.

## Introduction

Hip arthroplasty is now a standard procedure in the management of advanced degenerative hip joint changes [1–3]. The progress in operative techniques, combined with the use of high-quality implants, has made the procedure an easy process for the surgeon to perform and a fairly moderate condition for patient recovery [4–6]. All this has provided a basis for regarding this method as a treatment of choice even in young patients. In general, it is a corrective surgical procedure designed to restore normal function in the limb, eliminate pain and allow the patient unaided, effective mobility [7, 8]. The implant’s life span is one of the crucial problems in hip arthroplasty that depends on many factors, including the method of implant fixation in bone tissue, the type of joint articulation system used, the type of implant material and the option of implant surface finish with porous substances, facilitating the process of biological healing of the implant into bone tissue [9, 10]. The volume of microparticles, which are released from implant surfaces by friction during normal joint function, depends on the type of artificial joint surface finish [3, 11, 12]. Results of the latest studies have undoubtedly demonstrated that the ceramic-on-ceramic and metal-on-metal connections release the lowest volumes of microparticles during friction, which consequently reduces cytokine release, diminishing the risk for early loosening of implant components [13–15].

The goal of the study was to present the long-term results of cementless hip arthroplasty with ceramic-on-ceramic articulation.

## Materials and methods

Between the years 1985 and 1999, 220 Mittelmeier cementless hip endoprostheses were implanted in a consecutive series of 188 patients in our Department. In each case, an all-ceramic, threaded acetabular cup and a ceramic head of 32 mm diameter, placed on a steel stem (Autophor 900, model Mark II), were used. Since the year 1989, a new stem model has been implanted—Autophor 900S, model Mark III. Bilateral procedures were never performed during the same operation. The patients included 101 women and 87 men, with a mean age of 44.5 years (range 20–70 years). In 70 % the surgery was performed in patients below 50 years of age. The left hip joint was operated in 111 and the right in 109 cases. The mean observation period was 19.6 years (range 12.3–26.7 years). No patient died or was lost to follow-up. The aetiology of the disease in 81 cases (36.8 %) was associated with a history of hip dysplasia (dysplastic coxarthrosis) in childhood, idiopathic coxarthrosis in 60 cases (27.3 %), while other cases included traumatic background, aseptic necrosis of the femoral head and a history of childhood diseases (including juvenile arthritis). In two cases, patients with hip ankylosis were operated upon. The final results were evaluated by means of the Merle d’Aubigné and Postel classification, as modified by Charnley [16]. A detailed evaluation of the acetabular component implantation was performed by means of the DeLee and Charnley classification [17], while the stem implantation was assessed by the system of Gruen et al. [18]. Radiological evaluation also included geometric layout and axial location of the implants in the pelvis and the proximal end of the femoral bone. The implant’s life span was also evaluated by Kaplan-Meier estimators [19].

## Results

**Fig. 1:**
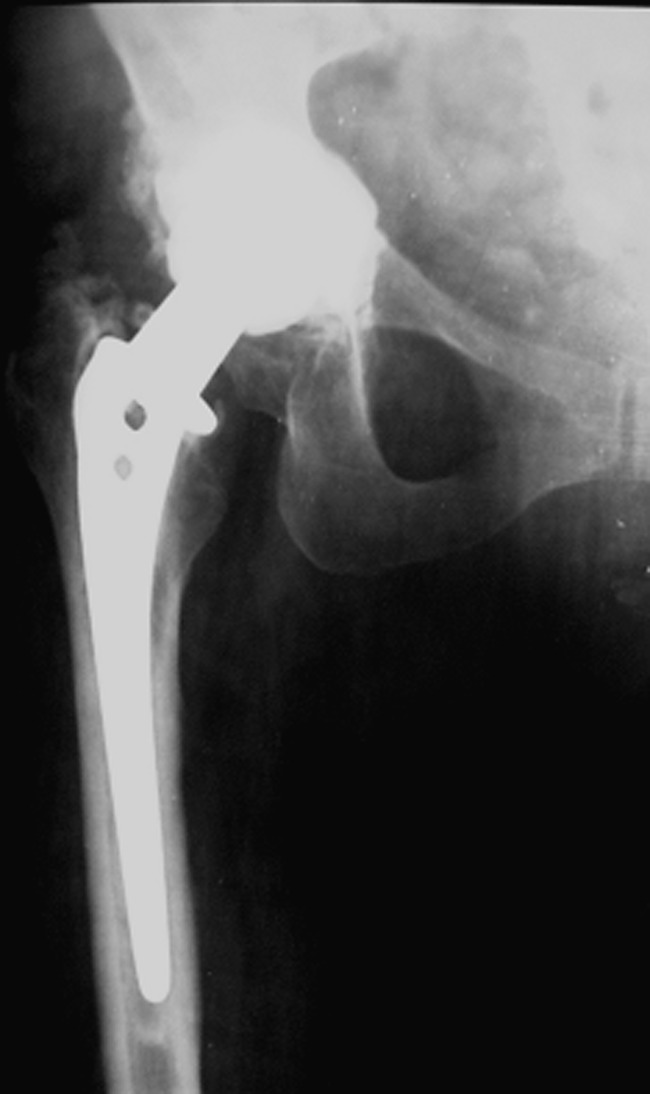
A 58-year-old patient 21 years after total hip replacement with a very good result

Very good results were obtained in 87 (39.5 %) of the patients, while in 96 cases (43.6 %) the results were assessed as good, in 20 cases (9.1 %) as satisfactory and in 17 cases (7.8 %) the surgical outcome was poor, achieving low scores in the Merle d’Aubigné and Postel classification (Fig. 1).

The very good and good results were mostly obtained in patients with idiopathic coxarthrosis. Both patients in whom a prosthesis was implanted after previous ankylosis presented with rather poor outcomes. The other poor results were mainly observed in cases of advanced dysplastic coxarthrosis, primarily classified as class IV and III according to Crowe et al.’s classification, and in patients with Autophor stem implants which, taking into account their smooth structure, demonstrated aseptic loosening. That group also comprised the highest number of complications, both intraoperative and late.

No joint squeaking was identified in any of the cases. Nor was there any trace of endoprosthesis infection in any of the cases.

Of the 220 Mittelmeier endoprostheses, 16 required revision surgery, including Autophor 900, type Mark II stem replacement in 11 cases, broken stem replacement (of unknown aetiology—most probably material fatigue) in cases and replacement of the acetabulum, broken in road accidents, in three cases.

Using Kaplan-Meier estimators, the following implant life span predictions were made: 12-year probability of whole prosthesis survival 86.36 %, 12-year probability of endoprosthesis acetabulum survival 89.99 % and 12-year probability of endoprosthesis stem survival 91.36 %.

## Discussion

The treatment of hip joint diseases and deformities in adult patients is one of the most challenging tasks in orthopaedics [1, 3, 20]. The first introduction of complete hip joint endoprostheses and their initial clinical applications 50 years ago started a new era of surgical hip joint replacement, solving—at least in part—the problems associated with hip joint failures. Arthroplasty provides patients with a chance to regain physical fitness and to return to social function within a relatively short time period after operation [3, 21]. However, implantation of artificial joints imposes a risk of wear and loosening or even breakage of endoprosthesis components over time [13, 22–25].

Systematic follow up studies and evaluation of surgical treatment outcomes allow us to evaluate the durability of the materials used to make the implants plus reactions and changes in the osseous tissue surrounding the endoprosthesis. An understanding of the pathophysiology of the osseous tissue around implants contributes—together with the development of the chemistry of plastics and metallurgy, materials science and technology and tribology—to continuous upgrading of implanted artificial joints to improve the outcomes of endoprosthetic replacement, especially in young and physically active patients [1, 3, 10, 21].

In the 1970s, a rapid development of cementless endoprosthesis implantations in bone tissue was observed. A number of biological bone-prosthesis bonding solutions were designed. Unlike cement which provides instant fixation, cementless prostheses require a certain time period for bone resorption, up to complete secondary, biological osseointegration. Taking into account the biology of osseous tissue, the intensity of which depends by and large on the age of patients, these endoprostheses are mainly applied in younger subjects with preserved osteogenic and reparative potentials and with good structural condition of both the acetabulum and the femoral bone. Mittelmeier’s model is an example of a cementless endoprosthesis [3, 11, 26, 27].

A Mittelmeier endoprosthesis was implanted for the first time in 1974 and was then frequently modified and improved for the entire 25-year period of its use [5, 7, 14, 27]. It consists of a ceramic acetabulum and a ceramic head with a metal stem. The shape of the acetabular component is that of a truncated cone, while its outer surface has a thick, intermittent thread, which ensures a solid, stress-resistant anchorage in bone. Processed ceramic elements present with outstanding smoothness of their surface, which guarantees optimal slipping properties, almost matching those of the natural hip joint of man. At first, the diameter of the ceramic head and the internal diameter of the ceramic acetabulum were 38 mm, to be then reduced to 32 mm. The femoral component is made of a metal alloy, including chromium and molybdenum, called “Endocast”, with an outer ribbing of type Mark I [5, 21, 26]. Following the rather high number of cases exhibiting instability with this type of stem, a stabilising “wing” and longitudinal grooving on the edges were added, and the collar base was corrugated in order to increase stem stability. The replacement of the transverse, relatively large ribs on the stem surface with oval, V-shaped cavities—Autophor 900 (Mark II)—was another, significant change. A cross-sectional image of the stem is rectangular with slightly rounded edges. Another—Autophor 900S (Mark III)—version was additionally covered with a porous structure over the whole surface [26]. We started using the Mittelmeier prosthesis at our Department with the Mark II model but later on, Autophor 900S, model Mark III stems were used exclusively, significantly improving the stability of the endoprosthesis stem.

Of the 220 implanted Mittelmeier endoprostheses, only 16 joints required revision operations for aseptic loosening of the Autophor 900 stem or mechanic failure of artificial joint elements. The majority of revision arthroplasty procedures involved the complex cases of dysplastic coxarthroses, necessitating additional procedures in the acetabular roof or in two cases of hip joint ankylosis.

Studies in recent years have demonstrated that osteolytic processes around the endoprosthesis are by and large associated with systemic reaction to the substances, released in the course of endoprosthesis wear [28]. These microcomponents, released during friction of artificial joint surfaces, induce biological, systemic reactions, leading to a release of proinflammatory cytokines from cells, surrounding the endoprosthetic elements. These cells additionally stimulate the secretion of metalloproteinases and influence osteoclasts, exerting a significant effect on the osteolytic processes. The activation of osteoblasts by cytokines, such as interleukin 6 (IL-6), or the stimulation of their differentiation by tumour necrosis factor alpha (TNF-α) enhances osteolysis around the implant [29, 30].

It seems that these reactions depend on the size and number of particles released from endoprosthetic surface friction. Thus, it is important to find a material for endoprosthesis elements which has the lowest friction coefficient. It appears from studies to date that this condition can be fulfilled exclusively by ceramic-on-ceramic systems. Studies carried out at several laboratories have indicated that the polyethylene abrasion rate is 0.01–0.3 mm/year, while it is merely 0.13–78 μm for ceramic joints [31]. Fewer particles from implant surface abrasion means smaller phagocytic reactions around the implant and a reduced incidence of endoprosthetic stem migrations, thus extending the endoprosthesis life span [29–31].

Yeung et al. reported outstanding results using an endoprosthesis with such articulation. The minimum ten year survival of endoprostheses was as high as 98 % [7]. Similar findings were published by Kress et al. In a minimum follow-up of ten years only one stem was loose among 65 arthroplasties performed [32]. Porat et al. compared the life span of cementless, ceramic-ceramic endoprostheses with metal-on-metal articulation systems. The per cent of loosening was 2.2 % in the former endoprostheses and 5.4 % in the latter systems [14]. In turn, D’Antonio et al. confirmed in their ten-year observation a much lower number of revision operations and cases of osteolysis using of implants with ceramic-on-ceramic articulation than with endoprostheses using metal-polyethylene articulation joints [15]. Hannouche et al. reviewed their 30 years’ experience with alumina-on-alumina bearings. They emphasise improvements in the fabrication and that the articulation is safe if the material is of high quality. Revision is easy to perform due to the lack of foreign body reaction and osteolysis [33].

An evaluation of radiographs, after Mittelmeier ceramic cup implantation, demonstrated increased radiotransparency areas in all zones, according to DeLee and Charnley’s classification, including almost one half of the implanted acetabulums (44.8 %). The width of those zones never exceeded one millimetre and was most pronounced in zone II. During subsequent control studies, the areas of increased radiotransparency were maintained with no change in size. That observation could be explained by the size of the acetabular thread base, which is much larger in the Mittelmeier ceramic acetabulum. Kody et al. [34], while studying the force of initial fixation of various acetabulum types, observed bone tissue crushing in the final stage of screwing in acetabulums with a large thread base. Moreover, the crushed bone layer separated from the remaining osseous tissue, which could be responsible for implant destabilisation.

The reports of other authors also indicate that in the case of ceramic implants, certain complications should be considered. Porat et al. provide worse outcomes with ceramic endoprostheses vs other cementless or cement endoprosthetic systems [14]. Mahoney and Dimon [24] report complications such as ceramic acetabulum breaks and fractures and Garcia-Cimbrelo et al. report worse results with the use of a Mark II stem [27]. Whittingham-Jones et al. also described a case of a broken ceramic acetabular component [13]. The majority of poor results, as in our material, resulted mainly from technically difficult operations in patients with orthopaedic history, in general, operated previously for hip disease in childhood. A technically correct operative technique during endoprosthesis implantation is also important since, following the observations, it is of key importance for the final outcome of the arthroplasty [33].

## Conclusions

Long-term results of hip arthroplasty using the Mittelmeier endoprosthesis are fairly encouraging, indicating a low percentage of loosened endoprosthetic components. The ceramic acetabulum heals very well into the bone bed, while the porous stem does not migrate or loosen. The degree of patient satisfaction after the Mittelmeier endoprosthesis is very high. Combined after proper indications, mainly regarding idiopathic coxarthrosis in young adults, it ensures long-term physical fitness and good clinical outcomes.

## References

[1] Heisel C, Silva M, Schmalzried TP (2004). Bearing surface options for total hip replacement in young patients. Instr Course Lect.

[2] Kim YH, Oh SH, Kim JS (2003). Primary total hip arthroplasty with a second-generation cementless total hip prosthesis in patients younger than fifty years of age. J Bone Joint Surg Am.

[3] Zwierzchowski H, Synder M (1992). Implantation der zementfreien Mittelmeier-Huftendoprothese bei Patienten nach Colonna-Plastik im Kindesalter. Orthop Praxis.

[4] Fenollosa J, Seminario P, Montijano C (2000). Ceramic hip prostheses in young patients: a retrospective study of 74 patients. Clin Orthop Relat Res.

[5] Mittelmeier H, Heisel J (1992). Sixteen-years’ experience with ceramic hip prostheses. Clin Orthop.

[6] Boehler M, Knahr K, Plenk H, Walter A, Salzer M, Schreiber V (1994). Long-term results of uncemented alumina acetabular implants. J Bone Joint Surg Br.

[7] Yeung E, Bott PT, Chana R, Jackson MP, Holloway I, Walter WL, Zicat BA, Walter WK (2012). Mid-term results of third-generation alumina-on-alumina ceramic bearings in cementless total hip arthroplasty: a ten-year minimum follow-up. J Bone Joint Surg Am.

[8] Capello WN (1990). Technical aspects of cementless total hip arthroplasty. Clin Orthop Relat Res.

[9] O’Leary JF, Mallory TH, Kraus TJ, Lombardi AV, Lye CL (1988). Mittelmeier ceramic total hip arthroplasty. A retrospective study. J Arthroplasty.

[10] Ivory JP, Kerschaw CJ, Choudhry R, Parmar H, Stoyle TF (1994). Autophor cementless total hip arthroplasty for osteoarthrosis secondary to congenital hip dysplasia. J Arthroplasty.

[11] Heisel J, Mittelmeier H (1993). Mittelfristige Ergebnisse der zementfreien Autophor-Hüftendoprothese. Z Orthop Ihre Grenzgeb.

[12] Engh CA, Hooten JP, Zettl-Schaffer KF, Ghaffarpour M, McGovern TF, Macalino GE, Zicat BA (1994). Porous-coated total hip replacement. Clin Orthop Relat Res.

[13] Whittingham-Jones P, Mann B, Coward P, Hart AJ, Skinner JA (2012). Fracture of a ceramic component in total hip replacement. J Bone Joint Surg Br.

[14] Porat M, Parvizi J, Sharkey PF, Berend KR, Lombardi AV, Barrack RL (2012). Causes of failure of ceramic-on-ceramic and metal-on-metal hip arthroplasties. Clin Orthop Relat Res.

[15] D’Antonio JA, Capello WN, Naughton M (2012). Ceramic bearings for total hip arthroplasty have high survivorship at 10 years. Clin Orthop Relat Res.

[16] Merle d’Aubigné R, Postel M (1954). Functional results of hip arthroplasty with acrylic prosthesis. J Bone Joint Surg Am.

[17] DeLee JG, Charnley J (1976). Radiological demarcation of cemented sockets in total hip replacement. Clin Orthop Relat Res.

[18] Gruen TA, McNeice GM, Amstutz HC (1979). “Modes of failure” of cemented stem-type femoral components: a radiographic analysis of loosening. Clin Orthop Relat Res.

[19] Kaplan EL, Meier P (1958). Nonparametric estimation from incomplete observations. J Am Stat Assoc.

[20] Crowe JF, Mani VJ, Ranawat CS (1979). Total hip replacement in congenital dislocation and dysplasia of the hip. J Bone Joint Surg Am.

[21] Mittelmeier H, Heisel J, Schmitt E (1988). Hüftgelenkersatz bei jungen Menschen unter 40 Jahren. Klinisch-statistischer Erfahrungsbericht. Z Orthop Ihre Grenzgeb.

[22] Della Valle AG, Becksaç B, Anderson J, Wright T, Nestor B, Pellicci PM, Salvati EA (2005). Late fatigue fracture of a modern cemented cobalt chrome stem for total hip arthroplasty: a report of 10 cases. J Arthroplasty.

[23] Yates PJ, Quraishi NA, Kop A, Howie DW, Marx C, Swarts E (2008). Fractures of modern high nitrogen stainless steel cemented stems: cause, mechanism, and avoidance in 14 cases. J Arthroplasty.

[24] Mahoney OM, Dimon JH (1990). Unsatisfactory results with a ceramic total hip prosthesis. J Bone Joint Surg Am.

[25] Fischer KJ, Carter DR, Maloney WJ (1992). In vitro study of initial stability of a conical collared femoral component. J Arthroplasty.

[26] Mittelmeier H (1986) Grundlage und allgemeine Erfahrungen mit dem Keramik-Prothesensystem Autophor/Xenophor. In: Mittelmeier H, Heisel (eds) 10 Jahre Erfahrungen mit Keramik-Hüftendoprothesen. Medizinische Literarische Verlaggeselschaft, Uelzen

[27] Garcia-Cimbrelo E, Martinez-Sayanes JM, Minuesa A, Munuera L (1996). Mittelmeier ceramic-ceramic prosthesis after 10 years. J Arthroplasty.

[28] Zywiel MG, Sayeed SA, Johnson AJ, Schmalzried TP, Mont MA (2011). State of the art in hard-on-hard bearings: how did we get here and what have we achieved?. Expert Rev Med Devices.

[29] Schmalzried TP, Callaghan JJ (1999). Wear in total hip and knee replacements. J Bone Joint Surg Am.

[30] Lassus J, Waris V, Xu JW, Li TF, Hao J, Nietosvaara Y, Santavirta S, Konttinen YT (2000). Increased interleukin-8 (IL-8) expression is related to aseptic loosening of total hip replacement. Arch Orthop Trauma Surg.

[31] Affatato S, Traina F, Toni A (2011). Microseparation and stripe wear in alumina-on-alumina hip implants. Int J Artif Organs.

[32] Kress AM, Schmidt R, Holzwarth U, Forst R, Mueller LA (2011). Excellent results with cementless total hip arthroplasty and alumina-on-alumina pairing: minimum ten-year follow-up. Int Orthop.

[33] Hannouche D, Zaoui A, Zadegan F, Sedel L, Nizard R (2011). Thirty years of experience with alumina-on-alumina bearings in total hip arthroplasty. Int Orthop.

[34] Kody MH, Kabo JM, Markolf KL (1990). Strength of initial mechanical fixation of screw ring acetabular components. Clin Orthop Relat Res.

